# Integrated structural equation modeling and causal steps in evaluating the role of the mediating variable

**DOI:** 10.1016/j.mex.2022.101777

**Published:** 2022-06-30

**Authors:** Muhammad Syafiq, Sirojuzilam, Badaruddin, Agus Purwoko

**Affiliations:** Universitas Sumatera Utara*,* Medan*,* Indonesia

**Keywords:** *Confirmatory factor analysis*, *Validity and reliability*, *Model modification*, *Regression analysis*, *Mediation analysis*

## Abstract

This study proposes an integrated analysis in evaluating the role of a mediating variable. An integrated method of structural equation modeling and causal steps was employed. The procedure commenced with the conceptual model and hypotheses development, followed by validity and reliability tests using International Business Machines (IBM) Statistical Package for the Social Sciences (SPSS) Statistics 25. Then confirmatory and regression analyses were carried out using Structural Equation Modeling (SEM)-Analysis of Moment Structures (AMOS), for model modification. Finally, the causal steps method was employed to evaluate the role of the mediating variable.

• This method integrates the use of SEM and causal steps
• It includes the validation of the model, variables, and constructs
• Also, it involves model modification

## Graphical abstract

**Figure:**
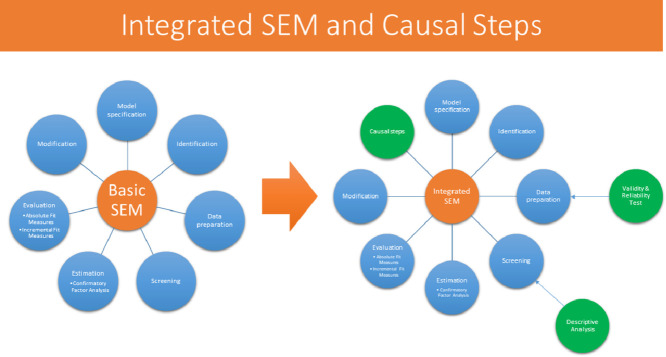

## Specifications table

**Table:** 

Subject Area	Environmental Science
More specific subject area	*Coastal development*
Method name	*Integrated Structural Equation Modeling and Causal Steps*
Name and reference of original method	*Structural Equation Modeling:* Weston, R., & Gore, P. A. (2006). A Brief Guide to Structural Equation Modeling. *The Counseling Psychologist, 34*(5), 719–751. 10.1177/0011000006286345
	*Causal Steps:* Kenny, D. A., Kashy, D. A., & Bolger, N. (1998). Data Analysis in Social Psychology. *In* D. Gilbert, S. Fiske, & G. Lindzey (Eds.), *The Handbook of Social Psychology* (4^th^ ed. pp. 233-265). Boston, MA: McGraw-Hill
Resource availability	*IBM SPSS Statistics 25:*https://www.ibm.com/support/pages/downloading-ibm-spss-statistics-25
	*IBM SPSS SEM-AMOS 26:*https://www.ibm.com/support/pages/downloading-ibm-spss-amos-26:
	*Da*ta: 10.17632/2ygw384v7m.1

## *Method details

Structural Equation Modeling (SEM) is a statistical tool for evaluating an established hypothesis concerning causal relationships among measurable and/or latent variables [14]. SEM is also a general approach to numerous statistical analytic investigations [2]. The capacity to explore the relationship between variables is one of its advantages over other quantitative methods [21].

In addition, Weston and Gore [21] summarized the six steps of SEM as follows: model specification, identification, data preparation, screening, estimation, evaluation, and modification. This study incorporated the six steps and used the causal steps approach as an extension, in evaluating the role of the intervening variable.

Combining SEM with the causal steps is not a completely novel concept which has been explored in several journal articles and books (Gunzler, et al, 2016; [4, 15]). However, the existing literature does not incorporate the preliminary analysis as part of a collective procedure. Validity and reliability tests, as well as descriptive statistics, are included in this study using IBM SPSS Statistics 25.

This approach involves several analyses namely:

a. Validity and Reliability tests
The Measuring equipment were put through Validity and reliability evaluation. A validity test determines how well the interpretations of results are supported by the evidence [12]. Reliability test examines an instrument's consistency in delivering the same finding when used repeatedly in similar scenario [5]
b. Descriptive analysis
Descriptive analysis is a statistical technique that aims to create a description of a phenomenon and its features [16]. Other types of statistics are also displayed, such as frequency distribution charts, histogram tables, mean and standard deviation values, etc. The benefit of this analysis is that it provides a comprehensive idea of the data being sought, whether it's in verbal or numerical form.
c. Confirmatory Factor Analysis
Confirmatory Factor Analysis (CFA) is a method for identifying and verifying the relationship between observed variables (such as test items, scores, and behavioural observation ratings) and latent factors [2].
d. Regression analysis
Regression analysis is a set of technique used to estimate relationships between two or more variables, and measure the "statistical significance" of the relations in comparison to the solid connection [18]
e. Mediation analysis
Mediation analysis is a method of determining the relevance of several pathways and mechanisms which determines the results [20].

### Model specification and identification

**Fig. 1:**
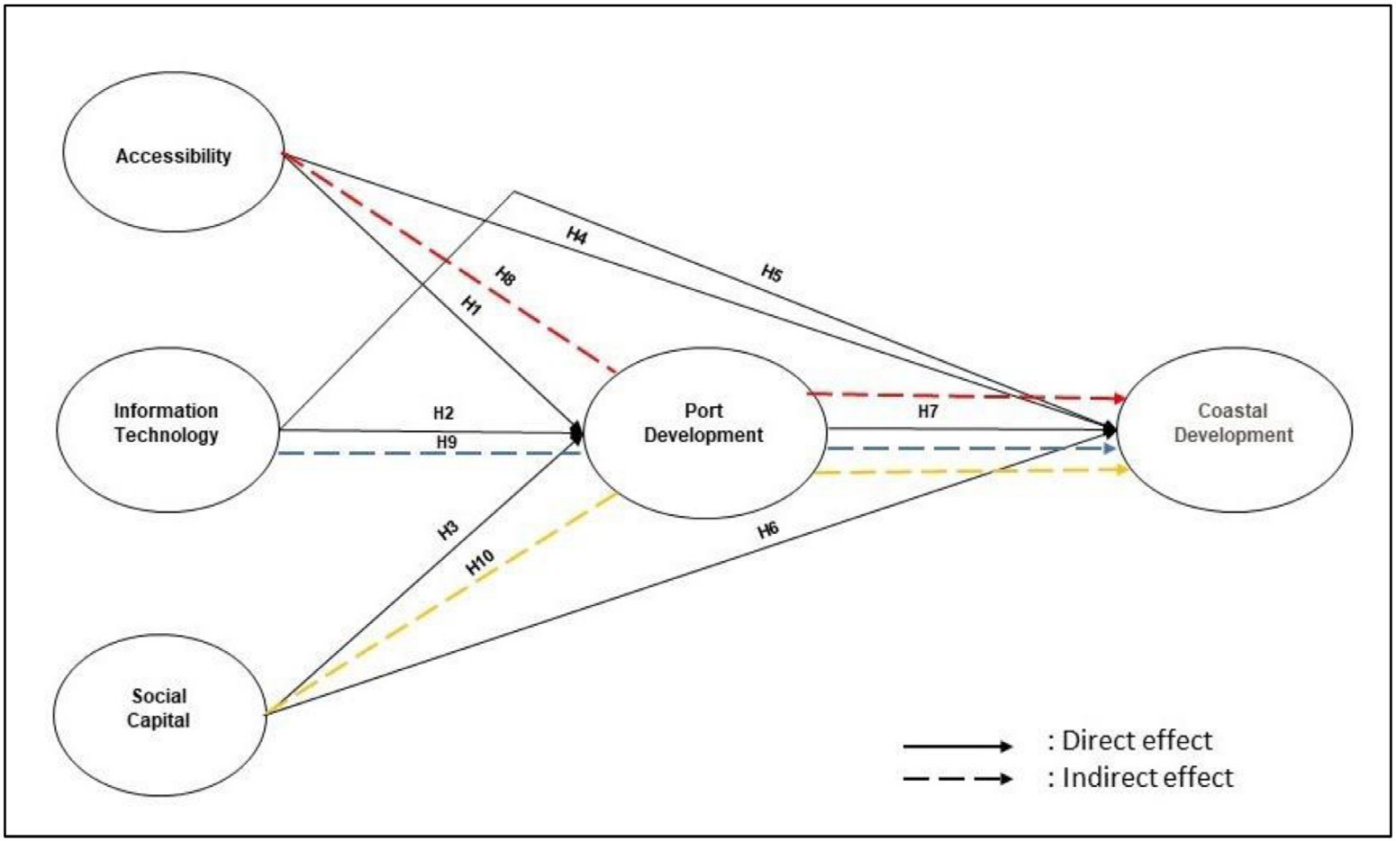
Conceptual model.

This stage encompassed the formation of a model, based on a theory or previous studies. This included the identification of the relationship between variables analyzed. The conceptual model is shown in Fig. 1.

Table 1 shows the Operational Definitions of the variables based on the conceptual model.

**Table 1:** Types of Variables and Operational Definition.

Types of Variables	Variables	Operational definition
Independent variables	Accessibility	Ease of getting the location
	Information technology	Use of technology in storing, managing and conveying data/information
	Social capital	Social values ​​that develop in the community
Mediating/intervening variable	Port development	Increasing the economic activities and capacity of the port
Dependent variable	Coastal development	Increasing the value of coastal areas on community welfare

.

The hypotheses were generated from the relationship provided in the conceptual model and based on preliminary studies. Hence, the proposed framework is used to derive these ten hypotheses (H1 to H10), namely:

H1: Port Development is influenced by Accessibility substantially and beneficially.
H2: The impact of Information Technology on Port Development is extensive and favourable.
H3: Port Development is influenced by Social Capital in a major and favourable manner.
H4: Coastal Development is controlled by Accessibility substantially and favourably.
H5: Information Technology influences Coastal Development in a favourable manner.
H6: Coastal Development is influenced by Social Capital substantially and beneficially.
H7: Port Development influences Coastal Development in an extensive and beneficial manner.
H8: The impact of Accessibility on Coastal Development is moderated by Port Development.
H8: Information Technology's impact on Coastal Development is mediated by Port Development.
H8: The impact of Social Capital on Coastal Development is moderated through Port Development.

### Data preparation and screening

This stage included the identification of samples and instrument validation. The number of samples was determined using the Slovin formula. Concerning the distribution ratio, the number of samples has to be proportionally divided considering the population in each area.

Additionally, the instrument used was a structured questionnaire using the Likert scale. The validation was carried out by testing the questionnaire on 30 respondents that were not part of the research sample [19].

Instruments validation involves validity and reliability tests using the Pearson correlation approach in IBM SPSS Statistic 25. The validity test was carried out to ascertain the feasibility of the questionnaire which was compiled in defining a variable. The survey is considered valid when it measures accurately, indicated by a higher r-value. Meanwhile, a reliability test was conducted to determine the consistency of the questionnaire. The survey is considered reliable when the answers obtained are consistent. Heale & Twycross [5] stated that Cronbach's is the most commonly used test to determine the internal consistency of an instrument. The questions that have more than two responses are used in this test. The Cronbach result is a defined number between 0 and 1, while the acceptable reliability scores are 0.7 and higher.

In addition, data were collected from questionnaires distributed to respondents, and the answers were given a certain value for the purpose of analysis: (a) assigned a score of 5, i.e., excellent, (b) = 4, i.e., good, (c) = 3, i.e., fair. The alternative answers were (e) = 1, i.e., terrible, (d) = 2, i.e., poor (Table 1).

Furthermore, descriptive analysis was conducted to evaluate the characteristics and answers of the research respondents regarding the analyzed variables. This assessment is critical to understanding the empirical condition which influences the results of the model analysis.

### Estimation

Exploratory Factor Analysis (EFA) and Confirmatory Factor Analysis (CFA) are the common statistical approaches for developing measuring instruments (Orcan, 2018; [17]). EFA is a factor method for determining the link between indicators while generating a structure [17]. When a research does not have the basic information, this approach is used to group series of indicators into variables, leaving the indicator and forming a parameter (Orcan, 2018). Whereas the CFA is used to evaluate the compatibility between the observational data and pre-conceptual, also the theoretically-based models that establish hypothetical causal relationships among their variables and observed indicators [13]. The primary distinction between the two analyses is that in CFA, there is an assumption that the indicators falls into a particular latent variable.

This study's hypothetical model was created using a theoretical framework of existing research. Hence, the measurement model was tested using Confirmatory Factor Analysis (CFA). Additionally, CFA is used to assess the indicators' ability in explaining a variable. It also assesses the validity and reliability of the instrument and the qualification of a variable as a good indicator. Therefore, the level of influence of an indicator in explaining a variable is determined in this analysis. An indicator is considered valid when it has a loading factor value greater than 0.3. All valid indicators were included in the full model test.

After the overall model was confirmed good, the research hypothesis evaluation was conducted using the estimated value and probability from regression analysis and SEM-AMOS. When the estimated value is negative, the independent variable has a reverse relationship with a mediating or dependent factor. Additionally, a positive value shows a unidirectional independent variable with mediating or dependent factors. Furthermore, the probability value that is lower than 0.05 indicates a significant influence of the variable over another.

### Evaluation of fit

The fit model was examined with several measuring instruments [9] which include

• Absolute Fit Measures (AFM): This examines the suitability of the structural and the measurement model. Some references in AFM were:
  a. Likelihood Ratio Chi-Square Statistics (LRCS), a large Chi-Square value on the degree of freedom indicates that the covariance matrix or the observed correlation is significantly different. This results in a probability (p) smaller than the significance level (α). However, a small Chi-Square value produces a (p) value that is greater than the (α) level. This indicates that the input covariance matrix between predictions and the actual observations is not significantly different. The Chi-Square value is expected to be insignificant, enabling the proposed model to correlate with the research data.
  b. Root Mean Square Error of Approximation (RMSEA) is a measure that improves the tendency of the Chi-Square statistics to reject models with large samples. RMSEA value is good when it is below 0.08.
  c. Goodness-of-Fit Index (GFI), is a measure whose value ranges from 0 to 1.0. No standard shows an acceptable GFI value as a rational ratio. A high GFI value indicates a better fit and a score equal to or greater than 0.90 is a measure of a good index.
• Incremental Fit Measures (IFM): This compared the proposed and the basic (null) model. Some of the references in this evaluation were:
  1) Adjusted Goodness-of-Fit Index (AGFI) is the alteration of GFI based on the degree of freedom for the proposed and the null model. The recommended value is equal to or greater than 0.90.
  2) Tucker-Lewis Index (TLI) is the combination of the measure of parsimony and the comparison between the proposed and the null model with TLI values ​​ranging from 0 to 1.0. While the recommended score is equal to or greater than 0.90.
  3) Comparative Fit Index (CFI) is a model feasibility test that is insensitive to sample size and model complexity, and it is highly recommended. The endorsed CFI value is equal to or greater than 0.90 [6].

### Model modification

Model modifications were employed to obtain a better type [3]. The basis for this modification encompasses Arbuckle's theory which recommends the connection of residuals from some of these indicators, for the model to fit the data, using the Modification Indices Table (MIT) guide [3].

Through this Modification Indices Table, several alterations were made, such as connecting the covariance between the models. This occurred for the model to meet the Goodness-of-Fit standard.

### Mediation analysis

Mediation analysis was carried out based on the regression assessment. A higher value of indirect effect than the direction of a variable on another indicates the existence of mediating variable roles. However, to determine the significance of the intervening variable in mediating the relationship between the dependent and the independent factor, the analysis was carried out with a causal steps approach. According to Baron & Kenny [1], Judd & Kenny [8], and James & Brett [7] in Kenny [10], there are four steps to determine the significance of mediation, namely:

1. Observing the effect of the independent variable (X) on the dependent (Z).
2. Exploring the impact of the independent variable (X) on the intervening variable (Y).
3. Observing the effect of the intervening variable (Y) on the dependent variable (Z).
4. Exploring whether the intervening variable (Y) fully mediates the effect of the independent variable (X) on the dependent (Z).

**Fig. 2:**
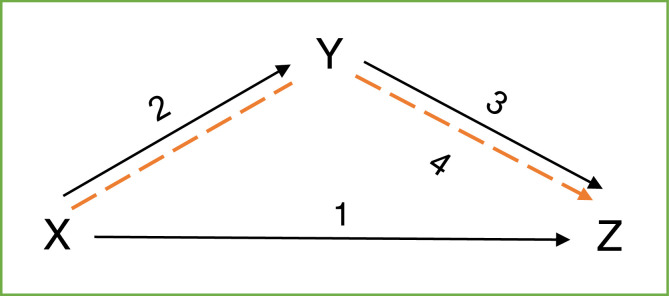
Mediational Model.

Figure 2 illustrates the mediational model of causal steps.

Additionally, steps 2 and 3 are the most important in studying the mediation of intervening variables. Step 4 is seldomly used until it is required to achieve full mediation, while stage 1 in the opinion of most analysts, is not necessary [11]. Although X to Z is insignificant, it is sufficient to prove the existence of mediation when the effects of X to Y and Y to Z are significant.

**Fig. 3:**
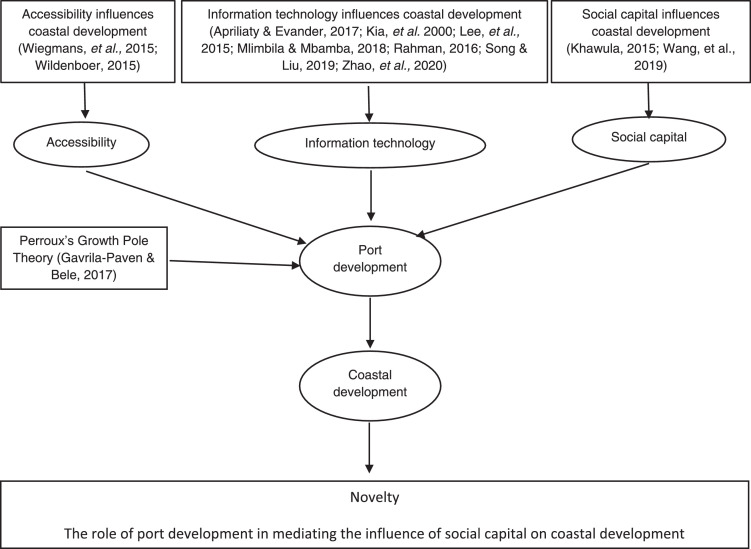
The method for novelty.

This study makes a scientific contribution to the field of coastal development and the significance of port development in mediating the effect of social capital as illustrated graphically in Fig. 3.

## Declaration of Competing Interests

The authors declare that they have no known competing financial interests or personal relationships that could have appeared to influence the work reported in this paper.

## Data Availability

I have shared the link to my data: https://doi.org/10.17632/2ygw384v7m.1.
